# Remedial, institutional or radical? Explaining community responses to violence against women in an NGO programme to prevent violence in Mumbai, India

**DOI:** 10.1016/j.worlddev.2024.106602

**Published:** 2024-07

**Authors:** Lu Gram, Sukanya Paradkar, Chatush Singh, Anand Suryavanshi, Beniamino Cislaghi, David Osrin, Nayreen Daruwalla

**Affiliations:** a Institute for Global Health, University College London, London, United Kingdom; b Society for Nutrition, Education and Health Action, Mumbai, India; c London School of Hygiene and Tropical Medicine, London, United Kingdom

**Keywords:** Violence against women, India, Community mobilisation, Social capital, Police

## Abstract

Despite ambitions in development and global health policy to transform communities into supportive environments for women facing risks of violence, our understanding of how to best engage communities remains incomplete. In particular, there is little evidence on the types of strategies that communities employ to address violence against women (VAW). We aimed to describe and analyse the processes involved in community responses to incidents of VAW in a non-governmental organisation (NGO) violence prevention programme in Mumbai, India. We conducted a grounded theory study involving 30 focus group discussions and 36 semi-structured interviews with 113 community members and 9 NGO staff, as well as over 170 h of field observation. Informed by comparative case study methods, we compared community actions across six informal settlement neighbourhoods. We found considerable variation in the type of action taken across neighbourhoods. This variation was not arbitrary, but reflected systematic cost-benefit considerations in heterogeneous environments, accounting for factors such as trust in neighbours, violent (armed or gang-related) crime, and corruption in state institutions. We found that institutional action was only favoured in neighbourhoods with strong state capacity and high social capital, whilst remedial action—resolving violence through ‘private’ talks in the family and community—and radical action involving extra-judicial violence—became favourable under conditions of weak social capital or low state capacity. Institutional action was, however, sometimes as violent as radical action, as police were reported, sometimes even relied upon, by residents to use force against perpetrators of VAW. We argue that these contextual features oblige policymakers and prevention researchers to grapple with contentious issues such as the legitimate use of force by the state. We caution against maximising community action without a clear vision of the *type* of action hoped for.

## Highlights

• We conducted a qualitative study of community responses to incidents of VAW in informal settlements in Mumbai, India.
• We identified three main strategies adopted by community members: remedial, institutional, and radical action.
• Social capital and state capacity shaped the favoured strategy in each neighbourhood, reflecting interveners’ cost-benefit considerations.
• Policymakers and practitioners need to consider what type of community action their interventions might elicit.
• We recommend intersectoral interventions promoting non-violence in both communities and state institutions.

## 1 Introduction

Globally, 27 % of women have experienced physical or sexual intimate partner violence in their lifetime (Sardinha et al., 2022). The United Nations Sustainable Development Goals have committed national governments to eliminate violence against women (VAW) by 2030, but it remains endemic in most of the world (UN, 2022). As well as being a profound development, public health, and human rights concern, VAW carries human, emotional, and economic costs for individuals and societies alike (Garcia-Moreno & Watts, 2011). Despite a growing body of evidence on interventions to prevent VAW (Jewkes et al., 2021), our understanding of effective intervention design remains incomplete and is evolving.

Community mobilisation—a holistic and participatory process in which communities challenge the broad social and institutional structures that perpetuate VAW (Honda et al., 2022, Minckas et al., 2020)—is recognised by policymakers as a critical approach to preventing VAW (Stern, 2021, USAID, 2020). Community action—individual or coordinated action taken by community members to challenge the social determinants of violence (Heise, 1998) and identify and support survivors (WHO, 2005)—remains a key pillar of this approach. In past programmes, community members have, for example, raised awareness, intervened in cases of violence, and referred survivors to community leaders (Lowe et al., 2022).

Inspired by ideals of participatory development in which local communities prioritise and decide on their own course of action to address shared challenges (Rosato et al., 2008), theories of community participation have often implicitly assumed that community action is positive (Gram et al., 2019, Minckas et al., 2020). Consequently, few studies have closely examined the choices made by community members in response to VAW. To address this gap, we present findings from a grounded theory study of community action to address VAW in a violence prevention program by non-governmental organisation (NGO) SNEHA (Society for Nutrition, Education and Health Action) operating in informal settlements in Mumbai, India. We sought to answer two key research questions: (1) What strategies do community members follow in responding^1^ to incidents of VAW in our setting? (2) How does neighbourhood context influence community members’ choice of strategy?

## 2 Literature review

Participatory interventions—in which external agencies collaborate with communities and stakeholders to address locally relevant challenges based on local expertise and input—have a decades-long history in global health and development (Mansuri & Rao, 2012). Community participation is said to counter power inequalities in top-down development programs which may produce locally unpopular, ineffective, or unsustainable projects (Mansuri & Rao, 2012). Interventions engaging community members in critical reflection and action to address VAW have been shown to be effective in Uganda and South Africa (Abramsky et al., 2014, Jewkes et al., 2021). However, large-scale trials of community-based interventions across South Asia have failed to show similar impacts, underlining a need to better understand intervention context and processes (Clark et al., 2020, Gibbs et al., 2020, Holden et al., 2016, Naved et al., 2018). An evaluation of women’s self-help groups—one of the most widely available platforms for bottom-up collective action among rural women in India—found little evidence for impact on domestic violence (Prillaman, 2023).

Multiple theories account for the role of communities in VAW prevention. Social disorganisation theory posits that crime emerges under conditions of social isolation and distrust due to an impaired collective ability amongst residents to regulate and control criminal behaviours (Sampson et al., 2002). Community members are thought to limit VAW by putting social pressure on perpetrators to stop, removing survivors from abusive situations, and connecting survivors to institutional services (Browning, 2002). The theory focuses particularly on the roles that concentrated disadvantage, residential instability, and ethnic heterogeneity play in eroding community solidarity and trust (Sampson et al., 2002). Social norms theory stresses the role of collective attitudes and expectations in sanctioning or mandating violence to uphold unequal gender roles (Gram et al., 2021). Community members prevent VAW by speaking out against violence and sexism, modelling non-violent relationships, and refusing to condone violent behaviour (Cislaghi et al., 2019, Daruwalla et al., 2019).

Existing theories of violence prevention have paid less attention to the *type* of action that communities might choose than to *whether* action is taken. They have largely assumed that a community member who seeks sincerely to address VAW—and does not outright victim-blame survivors or defend perpetrators—will engage in a variety of actions that contribute positively to VAW prevention, or at least do no harm (Abramsky et al., 2018, Lowe et al., 2022, Stern et al., 2021). Existing conceptual frameworks have also largely avoided theorising *why* different strategies might be chosen in different communities, leaving readers with a vague sense that well-intentioned community action perhaps only varies in relatively trivial ways across contexts (Mannell & Dadswell, 2017).

However, communities are known to respond to crime in quite varied ways, notably in interactions with state institutions such as the police (Tellez et al., 2020). Rule by village councils or chiefs is a well-known form of non-state crime control (Acemoglu et al., 2020, Cooper, 2018, Kumar, 2012), but less institutionalised forms of action such as extra-judicial violence have also been documented worldwide (Cohen et al., 2022, Sen and Pratten, 2007). In India, the Dalit women’s group *Gulabi Gang* (Pink Gang) in Uttar Pradesh are known for engaging in activities ranging from ‘beating up men who abuse their wives to shaming officials with whatever weapons are available including walking sticks, iron rods, axes, and even cricket bats’ (Dhillon, 2007).

As programme planners and policymakers seek to mobilise communities to address VAW, a better understanding of how communities make choices about what action they take is desirable. Two concepts may be helpful to achieve this. First, social capital—the aggregate characteristics of neighbourhoods which facilitate collective action, such as the presence of social networks, social cohesion, and interpersonal trust (Ostrom & Ahn, 2009)—may shape the degree to which residents expect support from neighbours in tackling cases of VAW. Second, state capacity^2^—the extent to which state institutions such as local government and the police are effectively governed and wield power locally (Cooper, 2018)—may shape the extent to which they believe in institutional solutions to VAW. In this paper, we will apply both concepts to describe and analyse communities’ choice of strategy to address VAW.

## 3 Context

India is a signatory to the Convention on the Elimination of All Forms of Discrimination Against Women (UN, 2008) and Indian law contains multiple provisions concerning VAW, most notably through the Indian Penal Code and the Protection of Women from Domestic Violence Act of 2005.^3^ Nevertheless, 22 % of women in India have experienced physical domestic violence, 7 % sexual domestic violence, and 22 % emotional or psychological violence in the past year (Kalokhe et al., 2017). India ranks 135 out of 146 countries on the Global Gender Gap Index, an international measure of gender inequality (WEF, 2022).

Our study took place in informal settlements in Mumbai. Gender norms often view managing the household and preserving family honour as central features of womanhood and justify domestic violence as a penalty for transgressing the husband’s wishes (Kostick et al., 2011). Many areas restrict female dress, mobility, and social interaction outside the home, creating public spaces that are visibly dominated by males (Cislaghi et al., 2020). Street sexual harassment, robbery, theft, and gang crime are common, but residents often refrain from seeking help from police who may ask for bribes (Subbaraman et al., 2014, Zietz and Das, 2018). Residents also tend to have fraught relations with law enforcement due to clashes over the legal status of their settlements, particularly during slum demolitions (Ghertner, 2008).

SNEHA runs a community-based programme to prevent VAW in this context, involving community engagement, counselling, and collaboration with institutional services (Daruwalla et al., 2015). Community engagement is implemented through facilitated group meetings and campaigns on gender issues with women, men, and adolescents. A cadre of male and female volunteers receive training to identify and support survivors through crisis intervention, referral to SNEHA counselling services, and help with access to health and police services. Counsellors support survivors through counselling, referral to mental health professionals, and collaboration with medical, legal, shelter, and police services (Daruwalla et al., 2019). SNEHA is not the only NGO providing services for survivors of violence in our context, but it is the largest provider in Mumbai.

## 4 Methods

The study was part of a broader grounded theory (Corbin & Strauss, 2014) study on community responses to incidents of VAW (Gram, Paradkar, Osrin, Daruwalla, & Cislaghi, 2023). We alternated between data collection and analysis, following an iterative loop of focus group discussions (FGDs) and semi-structured interviews (SSIs), observational fieldwork, data transcription and data analysis. We altered our sampling frame and topic guides continuously over the course of the study to explore new concepts identified in prior analysis. Our aim was to create rich, nuanced theory, capable of explaining variation in community responses to incidents of violence.

### 4.1 Sampling and recruitment

We sampled neighbourhoods,^3^ SNEHA-run community groups, and individuals based on theoretically relevant categories in ongoing data analysis (Corbin & Strauss, 2014). Any community member aged 18 or above was eligible to participate. We based our initial sampling on factors expected to influence willingness and ability to respond to incidents of VAW: degree of exposure to the SNEHA programme—whether general community member, current member of a SNEHA group, or community volunteer—and sociodemographic factors such as occupation, education, and religion (Abramsky et al., 2018). We asked SNEHA staff to identify particularly active or inactive SNEHA groups and sampled particularly vocal or silent FGD participants for follow-up SSIs. Follow-up SSIs allowed respondents to voice opinions or discuss personal experiences that they did not feel comfortable sharing with neighbours in an FGD setting. They also enabled us to delve deeper into key incidents that had only been raised briefly in FGDs.

Over the course of the study, we discovered that neighbourhoods varied markedly, not only in the level of action taken, but also in the type of action. We sampled neighbourhoods for additional FGDs and SSIs to investigate, focusing topic guides on concepts in need of refinement, such as community members’ decision-making processes for choosing a course of action. SNEHA staff recruited participants in person, or over the phone if they were already registered with a SNEHA group. We held focus groups and interviews with SNEHA staff to obtain their perspectives. To learn about communities in ways not possible in a formal interview setting, we conducted unstructured, non-participant observational fieldwork.

Table 1 here: List of neighbourhoods in which data collection took place.

**Table 1:** Main neighbourhoods in which data collection took place.

ID	No. of households	Majority religion	Migrant population?	Housing materials	Main occupation
A	1,000	Muslim	Yes	Plastic & tin	Carpentry & embroidery
B	850	Mixed*	Yes	Plastic & tin	Street vendors, sanitation workers
C	600	Hindu and Muslim	Yes	Concrete	Salaried employees
D	750	Mixed*	No	Concrete	Textile factory workers
E	800	Hindu	Yes	Concrete	Drivers, small business owners
F	700	Mixed*	Yes	Plastic & tin	Housekeeping & cleaning

*Note.* Housing made of concrete rather than plastic and tin indicates higher socio-economic status. * Hindu, Muslim, and Christian residents.

Data collection took place in 2021–2022 across two large informal settlements in Mumbai: Dharavi and Govandi. Table 1 lists the main neighbourhoods in which data collection took place.^4^ Each contained approximately 600–1000 households. SNEHA ran two to eight community groups in each neighbourhood. Residents were Hindu, Muslim, or Christian and all except one neighbourhood were migrant communities containing mainly first- and second-generation migrants from rural Maharashtra, Bihar, and Uttar Pradesh.

Table 2 here: List of focus group discussions with community members.

**Table 2:** List of focus group discussions with community members.

ID	Sex	Type	Expected activity level	Number of participants	SNEHA exposure	Neighbourhood
FG1	Female	General	N/A**	5	None	D
FG2	Female	General	N/A**	7	None	F
FG3	Female	Group members	Active	6	4 years	D
FG4	Female	Group members	Inactive	6	3–5 years	B
FG5	Female	Group members	Active	5	3 years	A
FG6	Female	Group members	Inactive	5	3 years	A
FG7	Female	Group members	Inactive	6	2–4 years	C
FG8	Female*	Group members	Active	8	3 years	E
FG9	Female	Volunteers	Active	7	1–15 years	D
FG10	Female	Volunteers	Active	5	8–14 years	E
FG11	Female	Volunteers	Inactive	4	3–7 years	C
FG12	Female*	Volunteers	Inactive	5	3 years	B
FG13	Female*	Volunteers	Active	6	1–6 years	F
MG14	Male	General	N/A**	5	None	D
MG15	Male	General	N/A**	5	None	F
MG16	Male	Group members	Active	4	4 years	†
MG17	Male	Group members	Inactive	7	1.5 years	†
MG18	Male	Volunteers	Active	5	2–13 years	†
MG19	Male	Volunteers	Inactive	5	1.5–4 years	†

*Note. ‘*Volunteers’ are community volunteers trained by SNEHA. ‘Group members’ participate in SNEHA-run group meetings in the community. ‘General’ refers to general community members who are neither volunteers nor group members. ‘Expected activity level’ refers to the degree to which SNEHA staff judged the group to be actively engaged in tackling VAW—rather than passive—at the stage of sampling. * Received a follow-up focus group discussion. ** SNEHA staff cannot judge activity levels of community members who do not join SNEHA events. † FGD members are from informal settlement neighbourhoods outside of A-F.

We did 16 FGDs with 75 women, six FGDs with 31 men, 19 SSIs with women, and 8 SSIs with men. Details of FGDs are provided in Table 2^5^ and details of SSI participants are in a Supplementary File. We included one male and one female respondent who had stopped participating in SNEHA activities to triangulate reports of people who were still involved with those of people who had left. We also conducted repeat FGDs and repeat SSIs with three groups of women and three women interviewees to elaborate on rich data they had provided. Apart from two male group members who declined to participate for lack of time, no other community members refused to participate. We conducted five FGDs and four SSIs with SNEHA counsellors, community organisers, programme officers, and a programme coordinator. In total, we spoke to 113 community members and 9 SNEHA staff.

### 4.2 Data collection

The author SP conducted FGDs and SSIs in Hindi or Marathi. FGDs and SSIs lasted between 30 and 90 min, were held online over Google Hangout or face-to-face, and were audio-recorded. To stimulate discussion, we occasionally asked participants to act out short skits, draw, or watch video clips. In the skits, participants acted out past experiences of being asked to assist survivors of violence. The drawing exercises asked participants to sketch or write the names of people close to them and explain why they mattered to them. The video clips involved short (2 min) scenes of couple fights escalating into violence (shouts, name-calling, or a slap). SP and two assistant translators transcribed 10 % of recordings into Hindi or Marathi before translating them into English. The author LG listened to audio recordings and read through transcripts in Hindi and English to check translation quality and discuss interview techniques with SP. The remaining recordings were translated directly into English.

Observational fieldwork was conducted by SP through visits to neighbourhoods to observe SNEHA staff ‘doing rounds’ (going from house to house and speaking to community members), holding community meetings, and implementing awareness-raising campaigns. SP spent over 170 h ‘hanging out’ in neighbourhoods, informally chatting to residents, and observing street and domestic interactions. We used an open-ended template directing SP to notice aspects of the physical environment, local people, and their conversations. SP wrote field notes on paper and transcribed them digitally at the end of the day. SP and LG held debriefing sessions after each FGD, SSI, and observational visit to discuss new concepts and plan further data collection. SP and LG continually adjusted topic guides based on ongoing data analysis, resulting in unique topic guides for each FGD and SSI.

Topic guides explored motivations behind different types of community response to incidents of VAW. We elicited stories of respondents witnessing gendered violence and asked them why they reacted as reported, what others thought of their response, and what role institutions (SNEHA, other NGOs, police, courts, local government) played. Given frequent references to SNEHA, NGO or police engagement, we probed community relations with these institutional actors in depth. We also examined residents’ general views on VAW, including attitudes and norms that might justify it or delegitimise intervention in cases of domestic violence. Topic guides for SNEHA staff covered the role of communities in SNEHA’s strategy for preventing VAW, SNEHA-community relations, and past experiences working with the police and courts.

### 4.3 Data analysis

Transcripts of FGDs, SSIs, and field notes were coded by SP and LG. We used the software tool MAXQDA 2020. Transcripts were memoed using analytical tools from grounded theory, followed by open, axial and selective coding (Corbin & Strauss, 2014).

Informed by comparative case study methods (Kaarbo & Beasley, 1999), we used codes to compare and analyse variation in neighbourhood response to incidents of VAW. We first examined codes by neighbourhood, looking for codes directly or indirectly connecting neighbourhood context to type of community response (within-case analysis). Next, we disaggregated codes for community characteristics and response to incidents of VAW by neighbourhood and looked for patterns linking response type to community characteristics (between-case analysis). We integrated ideas, concepts and relationships into a single ‘storyline’ to check for logical consistency.

To ensure analytic rigour, stimulate reflective analysis, and reduce cultural bias, SP and LG discussed interpretations and findings with each other, co-authors, and SNEHA staff almost daily throughout the study. This enabled us to question assumptions, consider surprising findings, and critically examine our own role in generating theory from data. SP called past interviewees on the phone to clarify statements. At the end of data analysis, we held a meeting with senior and junior SNEHA staff to discuss findings and receive feedback.

### 4.4 Researcher positionality

Given the critical influence of researchers’ social position, experiences, and beliefs on research processes and findings (Berger, 2015), we provide a brief statement of positionality. Three authors (including second author SP and last author ND) identify as female, four (including first author LG) as male. Four (including SP and ND) are employees of SNEHA based in Mumbai, India, while three (including LG) are academics based in the United Kingdom. Being male and based in a high-income country, LG had a degree of privilege that distanced him from the experiences of women living in informal settlements in Mumbai. LG addressed this by drawing on his experience living and working in South Asia, analogies to own lived experiences of racism, and epistemic humility in dialogue with local partners. The study itself directly answered a key desire by SNEHA to better understand the mechanisms of its violence prevention programme. SP occupied an intermediate position, as an Indian qualitative researcher and PhD student in political science with a privileged socioeconomic background compared to local respondents. However, social distance to respondents was muted by SP’s young age given locally prevalent hierarchies of age.

### 4.5 Limitations

Desires by study participants to favourably impress the interviewer may colour self-reports. We mitigated this risk by triangulating against field observations, discussions with SNEHA staff who routinely log community actions and cases of VAW in a central database, and interviews with community members who had never been involved in the programme or had left it. The cross-sectional nature of data collection meant that we relied on respondent recall in reconstructing changes in themselves and their communities over time, which is imperfect. Although new analytical insights became rarer towards the end of the study, some concepts remained unsaturated. In particular, we were limited in our exploration of the impact of social capital and state capacity on community action by a lack of neighbourhoods with high state capacity, but low social capital—the closest approximation was a neighbourhood with moderate state capacity, but low social capital (neighbourhood C). Even neighbourhoods with ‘high’ state capacity (D and E) only had high capacity relative to other neighbourhoods in our sample, as there are few examples of strong state capacity within Mumbai informal settlements in general. Finally, we restrained explorations into why social capital and state capacity varied across neighbourhoods in the first place to set reasonable boundaries on the scope of the study.

### 4.6 Ethics

Ethical approval for the study was provided by the Institutional Ethics Committee of Partners for Urban Knowledge, Action, and Research (PUKAR) in India (30 June 2021), and the University College London Research Ethics Committee (16603/001; 20 June 2021). We followed World Health Organization guidelines for research on domestic violence against women (WHO, 2001). We provided respondents participant information sheets and obtained consent. For face-to-face interviews, we obtained signed consent or thumb prints when they could not read or write. We took verbal consent for online interviews. We stored recordings securely and wiped them after transcription. In the transcriptions, all names and identifiers were replaced by pseudonyms. We also obtained informed consent for field observations from SNEHA staff and community members. When observation yielded personally sensitive information, we double-checked with staff and community members if they agreed to us recording it in our field notes in anonymised form. A protocol was followed for action in cases of disclosure of abuse or signs of distress from participants, including referral to services for survivors of violence.

## 5 Results

### 5.1 Perceived impacts of community engagement with SNEHA

Community members overwhelmingly indicated that participation in SNEHA activities had changed them and their neighbourhoods. They felt better informed about women’s rights, more capable of identifying gender inequality and domestic violence, and more confident in navigating interactions with the police or legal system. Many women reported a sense of empowerment from escaping the confines of their home, interacting with others in public, and standing up against abuse towards themselves:

“I became more astute, earlier I didn't know anything. I’d just get beaten up by my husband and take care of the children. That was all I knew … when I got out of the house, when I actually saw what was happening in the world outside, I became braver and realised that I wasn’t inferior to anyone. That I could do anything, I wanted to.” [FGD with female volunteers, FG10]

Respondents strongly emphasised becoming more active in supporting women facing violence after joining SNEHA. This involved becoming better at noticing incidents of violence, learning how to act and what to say, growing more confident and less fearful, and establishing an emotional commitment to addressing VAW. Residents remarked that they now talked about VAW where they had remained silent previously—“*Earlier, we never talked about these issues”* [interview with female group member, FI9]—and felt able to respond to incidents of violence instead of remaining powerless: “*Whenever we heard about an incident … we’d keep our thoughts about it to ourselves. We’d be angry, but we didn't really have the ability at the time to stop it then*” [FGD with male group members, MG17].

### 5.2 Community strategies in response to violence

While community members universally voiced greater confidence in their general ability to intervene after joining SNEHA, we found major variation in reports of how they acted in practice. We identified three major types of strategy adopted by community members in responding to incidents of VAW: remedial, institutional, and radical action.

#### 5.2.1 Strategy 1: Remedial action

The overall aim of this strategy was to resolve conflict and restore equilibrium to familial and community relationships with minimal disruption. VAW, particularly domestic violence, was thought to arise from insufficient tolerance and forbearance, mistrustful or unclear communication, and unfair allocation of household burdens: “*There is no capacity for tolerance. Among anyone. Everyone is just like this these days. They start shouting at tiny things that are amiss. It's normal at everyone's home*” [FGD with female volunteers, FG12].

Resolution was thought best achieved by involving as few people as possible to limit gossip and avoiding action that might escalate tensions and disputes or invite backlash. Seen through this lens, the involvement of police or women’s organisations was undesirable, as it escalated a ‘private matter’ into a public issue and involved potentially powerful ‘outsiders’ to the neighbourhood in the dispute. For example, one female group member unfortunately became the subject of extensive gossip in her neighbourhood after simply being visited by a SNEHA outreach worker [ethnographic fieldnotes]. Another male volunteer explained that his entire community had once boycotted SNEHA over a case of domestic violence because SNEHA staff had taken the survivor to the police despite objections from the community [ethnographic fieldnotes].

Instead, these community members preferred interventions such as scolding perpetrators, reassuring and helping survivors with practical needs, and asking couples to reconcile their disagreements.^6^ These responses could border on victim blaming, when residents insisted that ‘both sides’ were to blame and survivors needed to better ‘manage’ the anger of their husbands by shutting up rather than arguing. Although respondents did report cases of violence being resolved through this strategy, its long-term impact was unclear, as interveners witnessed cycles of violence flaring up, getting ‘resolved’, subsiding, flaring up, and worsening:

“They’d fight over tiny things. I’d go, reason with them, and get it resolved. Then again, after a few days, something would happen, and I had to go again, and try to reason with them, and it’d get resolved. That’s how the cycle continued … I must have tried at least 10 times! Later, when he started throwing her out of the house, I let her stay over.” [interview with female volunteer, FI16]

#### 5.2.2 Strategy 2: Institutional action

This strategy sought to rely on formal institutions such as SNEHA, other NGOs, local government, and the police to address survivors’ needs. Institutions were thought to have more resources, expertise, and authority than ordinary community members: “*If we can’t deal with it on our own, then we seek help from SNEHA, if not SNEHA, then the police. Then, big people will figure things out, no? We are small people*” [interview with female volunteer, FI17]. Another community member said that one should just take cases of domestic violence to the police, because “*it’s the police who can figure out how much of what was said was the truth and how much a lie. They’ve studied these things. They are more educated, and they’ve seen the world* … *they are naturals”* [Interview with female community member, FI2].

A key guideline was an emphasis on legal avenues of dispute resolution. For example, a volunteer had counselled a distressed mother whose daughter-in-law had run away with their grandchild, claiming that it was not actually theirs, to take a DNA test to prove paternity, so that ‘whatever needed to be done’ was ‘done legally’ [interview with female volunteer, FI17]. Community members following this strategy helped survivors register cases with SNEHA, other NGOs or the police, reported cases to authorities or asked them for help, helped police catch or collect evidence on offenders, or petitioned politicians for assistance.^7^ For example, a group of women had asked the police to speak to local men harassing women on the street: “*We went to the police station, brought them here, and got them to speak to them. It was then, that [the men] listened. They actually started begging us and the officers for mercy and said it wouldn't happen again!*” [FGD with female group members, FG8].

‘Institutional’ did not mean ‘non-violent’ as police were reported to use physical violence against perpetrators under arrest. Adopters of this strategy sometimes approved of institutional violence as legitimate and necessary: “*Shortly after marriage, the husband would hit his wife a lot* … *In the end, she registered a complaint against him with the police. The police arrested him, beat him up, and reasoned with him. He’s much improved now*” [FGD with female community members, FG1]. They might also threaten perpetrators themselves with police violence. For example, a volunteer explained that when dealing with abusive alcohol-using husbands, “*what we can do is to try to reason with them or threaten them like, ‘We will lock you up and the police will beat you and punish you’*” [interview with female volunteer, FI17]. Such behaviour was not the default, but did occur in some cases.

#### 5.2.3 Strategy 3: Radical action

This strategy entailed confrontation and often violence as a means of addressing VAW. It included threatening or carrying out physical attacks on perpetrators, liaising with local gangs to threaten perpetrators, telling survivors of violence to hit back at perpetrators, or participating in violent mass protest and property destruction. In extreme cases, it could involve collective violence. A volunteer described how a young man had had to be rescued by a local female gang when facing retaliation for harming a woman from an adjoining area:

“*Recently, a young man from our area went to a neighbouring area and sexually harassed a … woman or someone’s mother. He had also beaten her. Now, when you do something like that, do people just let you be? They came here carrying swords and all, ready to attack that boy … They were about to cut him—they had beaten him badly on hands and neck. These women rescued him. They were like, ‘How dare an outsider beat up someone in our area?’”* [interview with female volunteer, FI12]

Use of this strategy reflected a common belief that perpetrators only responded to credible threats of punishment. For example, one female volunteer cited the case of two sisters and their mother living next door who worked through the night for their household. When their father came home drunk and hit them and their mother, they hit him back. She explained, “*Now they are not kids who just tolerate the beatings. They don’t hesitate, they fight back properly and beat him up. Both women and children [do this], everyone* … *[In this age,] unless you are uncompromising, as a woman or a man, you will not be able to survive*” [FGD with female volunteers, FG12].

However, use of this strategy was also often motivated by concerns beyond the welfare of the survivor, such as the desire to achieve justice for survivors, challenge systemic corruption, penalise perpetrators for their behaviour, or exact revenge. For example, community members had engaged in disruptive protests over police inaction in cases where survivors had already died by either femicide or suicide [ethnographic fieldnotes].

### 5.3 Characterising neighbourhoods

**Figure 1:**
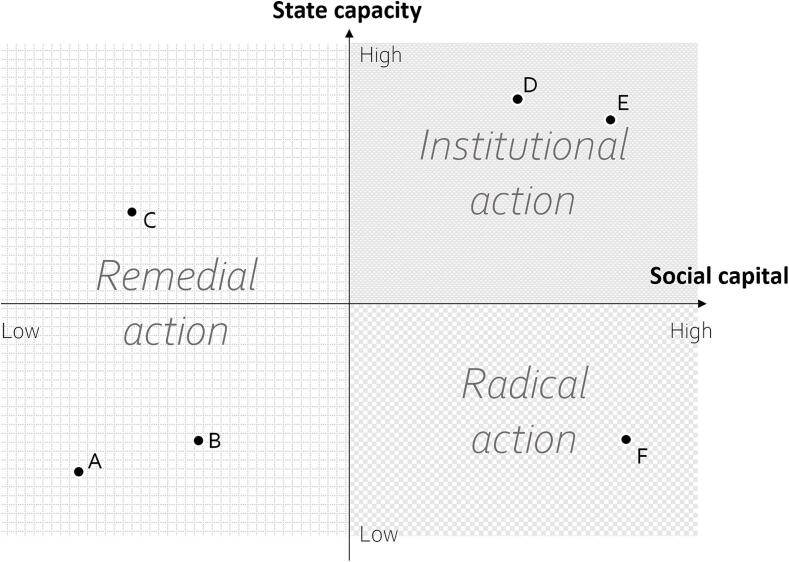
*Neighbourhood context and strategy choice Note*. Letters indicate approximate positions of neighbourhoods. Labels on quadrants indicate preferred strategies to address violence against women. For instance, both remedial and institutional action occurred in neighbourhood F, but radical action was the preferred strategy. Axes represent continua. For example, neighbourhood C has an intermediate degree of state capacity, but little social capital.

All three strategies were observed in most neighbourhoods and community members combined or switched strategies, when one was tried and found ineffective. In particular, community members often attempted remedial action before turning to institutional or radical action. However, over the course of SNEHA’s extended engagement with communities, residents’ responses had become routinised to the point at which the most active community members defaulted to one strategy. Comparison of neighbourhoods revealed that this choice varied meaningfully with context (Figure 1). Social capital separated neighbourhoods in which remedial action was the preferred strategy (A, B and C) from other neighbourhoods (D, E and F). State capacity distinguished neighbourhoods in which institutional action was the norm (D and E) from the one (F) in which radical action was favoured.

#### 5.3.1 Contexts favouring remedial action

All three neighbourhoods (A, B, and C) in which remedial action was the preferred strategy were characterised by a lack of social capital. Women’s social networks were restricted to family and relatives, with only superficial contact to neighbours. For example, one respondent exclaimed “*Now, where can one find friends these days!*” before specifying that her only close friend was her sister who lived four hours away by train. When asked about neighbours, she said, “*I don’t care about anybody in my neighbourhood*” [interview with female group member, FI6]. Rather than norms of cooperation and trust, community members often feared engaging neighbours they thought would be as likely to undermine them as help them: “*Madam, these days, no-one helps others … people talk behind your back, they laugh at you. There aren’t any nice people who don’t do that. There are many people who will come forward and say, “Let me help you out!” and instead sit and laugh at you*” [interview with female group member, FI10].

In two neighbourhoods (A and B), high levels of insecurity through crime and public violence indicated a lack of state capacity. In A, an interviewee described how a wave of robbery, murder, and violent sword crime had swept over the neighbourhood, exacerbated by COVID-19 lockdowns, to the extent that “*cutting people had become like cutting vegetables*” [interview with female volunteer, FI18]. Women feared graphic punishments for their son or husband if they spoke up, giving examples of past reprisal such as cutting a victim’s fingers off with a sword [ethnographic fieldnotes]. Police were noted primarily for their absence and visible lack of power, emblematised by the police outpost being razed to the ground by local drug users [ethnographic field notes].

In B, a criminal gang of women had established de facto rule ‘like a Don’ over the neighbourhood using threats, bullying, intimidation, and extortion. Citing a well-known Hindi proverb that ‘*whoever wields the club gets the ox*/jiski lathi usi ki bhains^8^’—roughly translating to ‘might makes right’—a volunteer explained that these women feared neither the police, whom they could ignore or bribe, nor political parties, of which they were already members, nor SNEHA or other NGOs, which held little authority in their neighbourhood [interview with female volunteer, FI12]. She explained that they ‘settled disputes’ between neighbours and family members through threats of violence in exchange for payment.^9^

C was a wealthier neighbourhood, whose respondent accounts indicated intermediate levels of state capacity. Local government, police, SNEHA and other NGOs were widely described as responsive and well-intentioned, whilst gang crime and pervasive violence were reported to be absent. However, public violence still occurred. Residents particularly complained of drunk men sexually harassing women and starting fights. In one instance, a man had peeped at the daughter of a female volunteer. When her son confronted him over it, he had threatened to kill him. Community members rarely reported such instances to authorities, fearing reprisal and believing the police unable to catch offenders anyway [ethnographic fieldnotes].

#### 5.3.2 Contexts favouring institutional action

Neighbourhoods in which institutional action was a common strategy (D, E) displayed signs of ample social capital. Residents reported a strong sense of community from communal festivals, informal socialisation, and cooperation based around an ethic of reciprocity: “*Everyone helps each other because, they think, ‘If you help me out today, then next time I will come to help you.’ And in this way, everyone’s tasks get done and everyone is happy about it*” [interview with female volunteer, FI13]. In contrast to other neighbourhoods who repeatedly declaimed against gang fights, alcohol dependency, and illicit drug use, respondents in D and E insisted that these were minor issues.

D and E also showed evidence of high state capacity. First, residents reported constructive relations with local government officials: “*I honour our councillor/*nagar sevak *and he honours me and my husband too*” [FGD with female volunteers, FG10]. Whilst other neighbourhoods were struggling with clean water, sanitation, or electricity, residents in D and E had moved on to (successfully) petitioning officials for public goods such as a skywalk over a commercial road, a bus service for commuting domestic workers, and streetlights and street cameras to deter theft. There was an ease to interactions with local government that was lacking in other neighbourhoods: “*We have the phone number of the councillor*/nagar sevak … *His name is H. We call up H in case of any trouble. The local contractor is R. R doesn’t listen to us. He listens to H … So, if R doesn’t answer, we tell H who comes and fixes things.*” [FGD with female volunteers, FG9].

Second, there was a broad consensus that the police were approachable and reliable. When asked if the police responded to phone calls in cases of VAW, a volunteer from D—who was herself married to a police officer^10^—responded, “*Yes, of course! They immediately come! They come in a big bus – our neighbourhood police are good, they support us.*” [interview with female volunteer, FI17]. Respondents gave many examples of police intervention, often in coordination with SNEHA and other NGOs who were respected for their access to the law. Tellingly, community members in D commended police officers for their bravery during COVID-19:

“During lockdown, many officers had died because of COVID-19. So how can we say that what they were doing was wrong? They themselves were battling the disease! God only knows what has happened to their children! So if they were stopping us from going out of the house, it was for our own benefit and well-being.” [FGD with female community members, FG1]

Other neighbourhoods had excoriated the police for their harsh COVID-19 lockdown measures, including beating residents breaking curfew, while residents of F had even thrown stones at passing police vehicles in response to these measures [ethnographic fieldnotes].

#### 5.3.3 Contexts favouring radical action

In neighbourhood F, radical action was a readily available strategy. Unlike A, B, and C, respondents in F reported strong social networks and high levels of cooperation, indicating high levels of social capital. They described themselves with idioms such as ‘living together like a fist/*ek mutthi ke jaisa*’, having a sense of ‘brotherhood/*bhaivaara*’, and ‘eating from the same plate/*ek bartan mein khaana khaate*’. They also had a history of community organising. In the past, they had clashed with police in large-scale protest when their water supply had been cut off, eventually forcing suppliers to restore the connection:

“Respondent 1: We closed off the highway to cause a traffic jam … People started running when the police suddenly charged at us with batons/laathi. They caught a boy from our area and so 50 of us women went to the police station [to support him] … The case against us lasted over six years. But our neighbours were really nice, they said that they had all taken part in the protest. They had participated because of us, and they should not let us suffer on our own.

Respondent 2: It’s like that, people here support each other in good times as well as bad. They don’t say that you are on your own with your problems.” [FGD with female community members, FG2]

However, low state capacity was evident in respondent accounts of local institutions, as these were pervaded by a sense that the police and local government were too corrupt to be reliable sources of help: *“In the garden over there, a handful of girls were raped by men who were high on drugs. There have also been murders … The police don’t do anything about these things. They take bribes and let the offenders go … The justice system here, the police, they are not on our side”* [FGD with male community members, MG14]. In a graphic case, the dead body of a 13 or 14-year-old girl was discovered in a pile of garbage by a rag picker, with burn marks all over her face and naked waist down. After multiple rallies to protest police inaction, CCTV footage revealed the rapist and killer to be a local Muslim cleric/*maulvi*. After a second woman was found murdered, residents rioted and vandalised the local bar [ethnographic fieldnotes].

Drawing on existing social capital, a SNEHA volunteer became a community leader, when she began supporting neighbours and friends facing violence ‘for free’ without taking payment. She said, “*Earlier, in my area, people had formed a women’s group, but they charged money to take up cases, about Rs. 5,000*–*10,000 … I don’t charge a single rupee from anybody, I hate money!”* [FGD with female volunteers, FG13]. Residents routinely approached her for help with disputes and were even encouraging her to run for office, for which she resolutely denied a desire [ethnographic fieldnotes]. As in neighbourhood B, female gangs ‘settled disputes’ through threats of violence in exchange for payment. Unlike in B, she was willing to use them to support women facing violence herself: “*[When] girls face eve-teasing [sexual harassment] in the alleyways, the police don’t listen to us. In these cases, it’s the goons that we go to and ask for help*” [FGD with female volunteers, FG13].

### 5.4 Mechanisms underpinning strategy choice

#### 5.4.1 Social capital

Social capital mattered because community members and SNEHA staff recommended acting collectively rather than alone. For example, a SNEHA officer explained how acting in concert provided witnesses in case of legal retaliation: “*People are wily. That’s why you need someone to support your claim and say, ‘This did not happen, this did!’”* [Interview with SNEHA staff]. Although remedial action did risk friction in relationships, radical action and institutional action carried greater risk because they were seen as more extreme. For example, a community member said, “*If a woman tries to retaliate by trying to hit [a sexual harasser] … but what if he has a knife on him? Are these alcoholics and drug addicts in their right senses?”* [interview with female community member, FI4]. Institutional action was also seen as unusual as it broke with traditional norms by escalating issues to a public level.

“If I had an issue at home, I would also think that I needed to keep my problems at home. But if it spilled out of our house and turned into a public spectacle, it’d be bound to end up involving others. Because everyone knows that people from that NGO come here to do social work. They work on women's issues.” [FGD with male group members, MG17]

Collective action was more likely to inspire confidence in interveners. Police and local government were often reported to dismiss complaints from individual women—even in neighbourhoods with greater institutional trust—unless survivors came with a group of neighbours or a second institutional actor, such as a SNEHA officer. A volunteer described repeated, dispiriting attempts at getting the police to act on bullying and harassment by a local gang without the support of others in the neighbourhood:

“When a proper group of women goes to the police station and demands action, then a lot of things can get done. But if you say, ‘Just go on your own, your issues will get dealt with!’ then nothing will happen, Ma’am, because the police don't listen! Has anyone trekked to the station and waited around/pulis ka chakkar kata as many times as I have? Spent as much money as I have spent, wasted as much time as me?” [FGD with female volunteers, FG12]

Radical action involving disruptive protest or threats benefited from large crowds, which were more intimidating than single individuals. In one case, a married woman had been forcibly locked in the attic for days by her in-laws. The family initially refused requests by neighbours to let her out, but after two volunteers gathered a crowd of over 50 people to protest her imprisonment and called both SNEHA staff and the police, the family got cold feet and negotiated a release with the rest of the community [interview with SNEHA staff]. Remedial action did not lend itself as easily to group action, as listening to survivors was best done in private [FGD with female group members, FG8].

#### 5.4.2 State capacity

State capacity influenced choice of strategy as timely responses to requests for help generated trust, whilst the opposite happened when police dismissed pleas or solicited bribes. People who had intervened in neighbourhoods with high state capacity voiced confidence in their helpfulness. For example, a volunteer in D had repeatedly tried to persuade a survivor to involve the police until she lost patience: “*The third time I went there [to stop a violent fight], I dragged her to the police station myself. I said, ‘Come, enough with this drama!’*” [interview with female volunteer, FI17]. In a different neighbourhood, a male group member had criticised the police for their slow or lacking response:

“People are already half dead by the time they get to the station. But once they reach the station, they lose all hope … the action is always delayed. And when it's delayed, then it demoralises the victim. Someone goes to lodge a complaint and you make them sit for four hours. The person is already hurt or battered. Fine, you have your ways of conducting an inquiry. But if you actually made the accused rather than the victim sit for four hours and cross-examine them, they would realise their wrongs.” [interview with male group member, MI23]

Weak state institutions also allowed crime and violence to flourish, making institutional action ineffective and potentially dangerous. A female volunteer in B said that neither police nor SNEHA could deter the criminal gang of women in her neighbourhood, “*To them, the police are nothing. The police have no power over them … SNEHA doesn’t hold any value for them [either]*” [interview with female volunteer, FI12]. Institutional action was seen as challenging their rule, leading to violent reprisal. In one case, gang members had stopped people from signing a petition to clean up local gutters organised by SNEHA, declaring, “*Whatever we say goes around here. Unless we’ve explicitly allowed something to happen, it can’t happen. If we say it’s day, then it's day. If we say it’s night, then it's night!*” [interview with female volunteer, FI12]. Remedial action to support survivors avoided backlash as it did not visibly challenge their authority.

Finally, weak institutions raised questions among community members over whether justice was really achieved through institutional action. This was starkly demonstrated when SNEHA staff tried to encourage community engagement with police in neighbourhood F. SNEHA held meetings at the police station to discuss domestic violence law with the community. A local volunteer was frustrated that illegal gambling was being openly practised with the police turning a blind eye: “*All the poor people who played, he robbed everyone of their money.*” [interview with female volunteer, FI14]. When an officer came to talk about ways to report corruption to the police, she stood up and objected, “*You’re the ones taking bribes! Shut down these shady businesses!*” [interview with female volunteer, FI14]. When members of the gambling ring next sexually harassed a fellow group member, she responded with violence:

*“They eve-teased [sexually harassed] my friend … I went and called on him. I confronted him and said, ‘Hey you! What did you say to her?’ He said he hadn’t said anything. I said, ‘Is she lying then?’ Then I slapped him a few times. I and one of my group members were there so we both hit him. My brother-in-law also held him and hit him … I said, ‘Do not set up your business here from tomorrow!’”* [interview with female volunteer, FI14]

## 6 Discussion

We have described and analysed the social processes involved in community responses to incidents of violence in a community mobilisation intervention to prevent VAW in urban India. Contrary to past conceptualisations of the role of communities in violence prevention (Gram et al., 2019), we found that community strategies to address VAW varied substantially with context. Social capital and state capacity shaped residents’ preferred choice of action through differential expectations of benefit and risk. Neighbourhoods lacking in social capital engaged primarily in remedial action as residents felt insufficiently protected from reprisal to escalate matters to the institutional level or take the law in their own hands. Residents of neighbourhoods with ample social capital and state capacity took institutional action as they felt collectively empowered to make institutions act in their interest. At the intersection of strong social capital and weak state institutions, residents took radical action, having the collective means to act, but not the motivation to work with corrupt institutions.

By accounting for variation in types of community action, our findings fill a gap in extant theories of community prevention of VAW. Social disorganisation theory bundles all community actions to prevent VAW into a unitary concept and measure called informal social control and does not theorise different types of social control (Browning, 2002). Social norms theories have similarly been less concerned with *how* communities uphold or punish violent behaviour than *whether* sanctions are applied or expected at all (Gram et al., 2021). Our findings indicate that community action should not be treated as a unitary construct. The types of actions taken by community members vary substantially and systematically, likely resulting in distinct consequences for perpetrators and survivors across neighbourhoods. For example, registration of domestic violence as a crime with the police enables subsequent legal action, but privately mediated reconciliation does not.

Our findings also bring together multiple perspectives on community action, law enforcement, and vigilantism. Our findings on residents’ use of remedial action agree with international and domestic evidence on survivor experiences of seeking help (Dworkin et al., 2019, Jacob and Chattopadhyay, 2019). The underlying aim of this strategy of restoring equilibrium to relationships through ‘private’ mediation makes sense in the light of a ‘logic of reconciliation’ in which survivors want above all to continue running a family together with perpetrators (Roychowdhury, 2020). The moral ambiguity of neighbourly advice has also been seen in statements by community members in Rwanda, who counselled women in abusive dating relationships to marry partners to improve their social and legal status (Mannell et al., 2018). While the above literature has characterised preferences for private resolution of VAW as responding to cultural norms, we argue that weak social capital also plays a role in making them attractive.

Echoing past studies in India, we found entrenched barriers to institutional action, including patriarchal beliefs among police officers, corruption and potential collusion with perpetrators based on bribes, and fraught relations between communities and the police (Jacob and Chattopadhyay, 2019, Menon and Allen, 2018, Panchanadeswaran and Koverola, 2005). Our findings suggest that these barriers can be mitigated, as we encountered neighbourhoods high in social capital and state capacity in which institutional action was routinely sought. This was no trivial task, however, as women only expected action after securing significant social and organisational backup. Roychowdhury’s (2020) findings on survivors’ experience of the Indian criminal justice system resonated profoundly:

“To engage the law and not be suppressed, women had to be cunning, socially connected, able to gather evidence, able to ferry documents, able to round up witnesses, aggressive, persuasive, and potentially violent both against the forces of law and against their own abusers.” (Chapter 1, p. 14).

Political scientists have long argued that non-state actors emerge to fill gaps left by weak states that fail to supply basic services to citizens (Acemoglu et al., 2020). Given well-known precedents of vigilantism to enforce religious, gender, and political ideology (Ahuja, 2019, Jaffrey, 2021, Sen, 2018), it was perhaps not unthinkable that radical action emerged as a potential strategy to address VAW in our context. Nonetheless, to our knowledge, only one study has previously described and analysed similar action to address VAW in India, namely White & Rastogi (2009)’s study of the vigilante women’s group *Gulabi Gang* in rural Uttar Pradesh—a very different context to urban Mumbai. White & Rastogi (2009) theorised that extra-judicial violence arises under conditions of structural injustice, normalised everyday violence, and collective victimhood. We extend their findings by noting the need for a conjunction between weak state capacity and strong social capital to trigger radical action.

Importantly, our findings problematise straightforward binaries between state and non-state action. Past literature has framed non-state action as ‘mob vigilantism’ (Wilke, 2023) or ‘violent justice-seeking’ (White & Rastogi, 2009). However, ‘mob vigilantism’ implies a degree of unthinking reactiveness that fits poorly with the fact that residents’ choice of action reflected rational cost-benefit considerations in different risk environments—a point often made by crowd psychologists (Drury, 2002). ‘Violent justice-seeking’ suggests that non-state actors are more violent than state actors, when police were reported, sometimes even relied upon, by community members to punish perpetrators with violence.^11^ Sundar (2010) perceptively points out the difficulties of drawing clear boundaries in India, when political parties and the state itself are known to have supported vigilante violence.

The social processes described in this paper are important to consider in programme design and policy. Whether remedial, institutional, or radical, community members’ choice of strategy to address VAW arguably reflects and expresses value judgments on power structures, gender norms and state institutions. Policymakers must take a stance on acceptable forms of community action, as their own interventions will likely elicit community actions falling within our typology. If non-violent institutional resolutions to cases of VAW are desired, intervention designers must simultaneously strengthen social capital and state capacity *and* promote non-violent, anti-oppressive practices in both communities and state institutions. Achieving this in informal settlement contexts will be no mean feat and will require truly intersectoral interventions bringing together community members, health and social services, and the criminal justice system to end VAW.

## CRediT authorship contribution statement

**Lu Gram:** Conceptualization, Formal analysis, Funding acquisition, Investigation, Methodology, Supervision, Writing – original draft, Writing – review & editing. **Sukanya Paradkar:** Formal analysis, Investigation, Project administration, Writing – original draft, Writing – review & editing. **Chatush Singh:** Investigation, Project administration, Validation. **Anand Suryavanshi:** Investigation, Project administration, Validation. **Beniamino Cislaghi:** Supervision, Writing – review & editing. **David Osrin:** Project administration, Supervision, Validation, Writing – review & editing. **Nayreen Daruwalla:** Project administration, Supervision, Validation, Writing – review & editing.

## Declaration of competing interest

The authors declare that they have no known competing financial interests or personal relationships that could have appeared to influence the work reported in this paper.

## Data Availability

Data will be made available on request.

## Footnotes

1 Community action to address VAW involves a broader range of behaviours than action taken in response to specific incidents of VAW. Community members may, for example, participate in awareness-raising activities concerning VAW without personally knowing of any incidents of VAW in their neighbourhood. We focus on community members’ responses to incidents of VAW, because our theory of change puts strong emphasis on secondary prevention—visibly supporting and responding to survivors’ needs as a pathway towards primary prevention—encouraging non-violent behaviour in the first place (Daruwalla et al., 2019).

2 Our conception of state capacity here is somewhat narrower than its usage in political science and international relations, in which the term often refers to the implementation power of national government as a whole (Lindvall & Teorell, 2016). We follow precedent set by Cooper (2018) who focused on their definition of state capacity on local security forces in Papua New Guinea, such as village police.

3 Civil law addresses domestic violence through the Protection of Women from Domestic Violence Act of 2005, while criminal law addresses it through Section 498-A of the Indian Penal Code (IPC). Sexual harassment at work is addressed by the Prevention of Sexual Harassment at Workplaces Act, while sexual harassment in public places is addressed in Sections 453 and 509 of the IPC. Sections 375 and 376 of the IPC address non-marital rape, but explicitly exempt marital rape.

4 For sampling purposes, we defined ‘a neighbourhood’ as a collection of people and institutions occupying a spatially delimited area influenced by shared ecological, cultural and political forces (Sampson et al., 2002). In practice, this meant that each ‘neighbourhood’ represented one or two wards at the corporator level.

5 Due to the iterative nature of grounded theory research, the degree of variation in community strategies to address VAW between neighbourhoods only became apparent later in the data collection process. As such, some earlier FGDs and interviews took place in neighbourhoods outside the ones in Table 1. We used these data to understand the broader context in which community action took place.

6 Community members who encouraged couples to separate, organised permanent shelter for survivors, or held public mediation sessions between survivors and perpetrators’ families went beyond the philosophy of remedial action, as these actions disrupted existing family and community relationships. However, their actions were not inspired by faith in institutions or a desire to overcome unjust systems. As such, they fall somewhat ‘in between’ the strategies in our typology.

7 Note that institutional action existed on a continuum: from doing the bare minimum by, for example, leaving the phone number of local authorities or mentioning the existence of services available from SNEHA or other NGOs to actively ensuring that perpetrators and their families show up to meetings with service providers and following up with survivors.

8 The proverb is commonly told as a children’s story in which an ox-herder is waylaid by a robber wielding a large club, demanding his ox. The herder agrees, but says that, at the very least, the robber could give him his club in return, as the ox is far more valuable than the club. The robber agrees to the exchange. However, as soon as the herder gets the club, he threatens the robber using the club and gets back his ox. The moral is that “whoever wields the club gets the ox.”.

9 Although women in neighbourhood B engaged in violence outside the boundaries of law, we did not classify this neighbourhood as choosing radical action as a solution to VAW. First, community members had not reported any actual cases of women being called upon to address VAW. Second, the women did not wield violence with the aim of preventing VAW or securing justice for survivors, but simply beat up whomever they were paid to attack. A volunteer remarked that, if a perpetrator paid them more than a survivor, they would happily attack the survivor [interview with female volunteer 3].

10 This respondent might have been motivated to provide more positive accounts of the police as she was married to a police officer. To avoid findings in this neighbourhood depending overmuch on her account, we triangulated her views with those of other respondents from her neighbourhood who overwhelmingly, independently voiced the same views. Note that her husband worked outside the neighbourhood as police officers regularly rotate between duty stations. She herself did not hesitate to voice criticisms of the police, such as their use of violence against perpetrators.

11 In the wake of Black Lives Matter, abolitionist feminists have also heavily criticised traditional feminist views on the use of the legal system to protect victimised women (Whalley & Hackett, 2017).
