# Supplementary material for: Remedial, institutional or radical? Explaining community responses to violence against women in an NGO programme to prevent violence in Mumbai, India
Source: World Dev. 2024 Jul;179:106602. doi: 10.1016/j.worlddev.2024.106602 (PMC11750413; doi:10.1016/j.worlddev.2024.106602)
Supplement: Supplementary Data 1 [file mmc1.docx]

**Table S1**

*List of semi-structured interviews with community members*

| **ID** | **Sex** | **Type** | **Age** | **Marital status** | **Religion** | **Education** | **Occupation** | **Neighbourhood** | **Interview mode** |
| --- | --- | --- | --- | --- | --- | --- | --- | --- | --- |
| FI1 | Female | General | 26-35 years | Married | Muslim | 5^th^ grade | Homemaker | D | Face to face |
| FI2 | Female | General | 56-65 years | Widowed | Muslim | 12^th^ grade | Homemaker | D | Face to face |
| FI3 | Female | General | 56-65 years | Married | Muslim | <5^th^ grade | Stitching work | F | Face to face |
| FI4 | Female | General | 26-35 years | Married | Hindu | 4^th^ grade | Homemaker | F | Face to face |
| FI5 | Female | Group member | 36-45 years | Married | Muslim | None | Homemaker | D | Face to face |
| FI6 | Female | Group member | 36-45 years | Married | Hindu | 8^th^ grade | Electrical parts | B | Face to face |
| FI7 | Female | Group member | 36-45 years | Married | Hindu | 10^th^ grade | Homemaker | E | Face to face |
| FI8 | Female | Group member | 36-45 years | Married | Muslim | <5^th^ grade | Homemaker | A | Face to face |
| FI9 | Female | Group member | 36-45 years | Married | Hindu | 12^th^ grade | Teacher | C | Face to face |
| FI10* | Female | Group member | 36-45 years | Married | Muslim | <5^th^ grade | Homemaker | A | Face to face |
| FI11 | Female | Volunteer | 36-45 years | Married | Muslim | 8^th^ grade | Homemaker | D | Face to face |
| FI12 | Female | Volunteer | 36-45 years | Married | Hindu | 10^th^ grade | Beauty parlour | B | Face to face |
| FI13 | Female | Volunteer | 36-45 years | Married | Muslim | 8^th^ grade | Homemaker | D | Face to face |
| FI14* | Female | Volunteer | 46-55 years | Married | Hindu | 7^th^ grade | Shop owner | F | Online |
| FI15 | Female | Volunteer | 26-35 years | Married | Muslim | 5^th^ grade | Stitching work | A | Online |
| FI16 | Female | Volunteer | 36-45 years | Married | Muslim | 7^th^ grade | Homemaker | C | Face to face |
| FI17* | Female | Volunteer | 36-45 years | Married | Hindu | 8^th^ grade | Stitching work | D | Face to face |
| FI18 | Female | Volunteer | 36-45 years | Married | Muslim | <5^th^ grade | Homemaker | A | Face to face |
| FI19 | Female | Ex-volunteer | 20-25 years | Married | Muslim | 9^th^ grade | Homemaker | C | Face to face |
| MI20 | Male | General | 20-25 years | Unmarried | Hindu | 12^th^ grade | Sales | F | Online |
| MI21 | Male | General | 36-45 years | Married | Muslim | 5^th^ grade | Welding | F | Online |
| MI22 | Male | Group member | 20-25 years | Unmarried | Hindu | Bachelor’s | Banking | † | Online |
| MI23 | Male | Group member | 26-35 years | Married | Muslim | 8^th^ grade | Transport | † | Online |
| MI24 | Male | Volunteer | 46-55 years | Married | Hindu | 10^th^ grade | Driver | † | Face to face |
| MI25 | Male | Volunteer | 20-25 years | Unmarried | Buddhist | Bachelor’s | Municipal worker | † | Face to face |
| MI26 | Male | Volunteer | 26-35 years | Unmarried | Hindu | Bachelor’s | Unemployed | † | Online |
| MI27 | Male | Ex-volunteer | 56-65 years | Married | Buddhist | 9th grade | Garments | † | Online |

*Note. ‘*Volunteers’ are NGO-trained community volunteers. ‘Group members’ participate in NGO-run group meetings in the community. ‘General’ refers to general community members who are neither volunteers nor group members. * Received a follow-up interview. † Respondent is not from any of A-F.
